# Comparative Analysis of Traditional Oriental Herbal Fruits as Potential Sources of Polyphenols and Minerals for Nutritional Supplements

**DOI:** 10.3390/molecules28062682

**Published:** 2023-03-16

**Authors:** José Javier Quesada-Granados, José Ángel Rufián-Henares, Suryakant Chakradhari, Pravin Kumar Sahu, Yaman Kumar Sahu, Khageshwar Singh Patel

**Affiliations:** 1 Departamento de Nutrición y Bromatología, Instituto de Nutrición y Tecnología de los Alimentos (INYTA), Universidad de Granada, 18071 Granada, Spain; 2 Centro de Investigación Biomédica, Instituto de Investigación Biosanitaria ibs.GRANADA, Universidad de Granada, 18071 Granada, Spain; 3 School of Studies in Environmental Science, Pt. Ravishankar Shukla University, Raipur 492010, India; 4 School of Studies in Chemistry, Pt. Ravishankar Shukla University, Raipur 492010, India

**Keywords:** herbal seeds, fruit seeds, polyphenols, minerals, nutritional supplements

## Abstract

There are a plethora of plant species in India, which have been widely used in vegetable dishes, soups, desserts and herbal medicine. In addition to these traditional uses, today there is the extra possibility of also being able to use these plants in the nutritional supplements industry due to their favorable antioxidant and mineral composition. In this sense, thirteen vegetable species—*Chanania lanzan*, *Ziziphus mauritiana*, *Nilumbo nucifera*, *Terminalia catappa*, *Terminalia arjuna*, *Terminalia bellirica*, *Terminalia chebula*, *Lagenaria siceraria*, *Luffa aegyptiaca*, *Praecitrullus fistulosus*, *Benincasa hispida*, *Citrullus lanatus* var. lanatus and *Cucurbita maxima*—have been analyzed. In this paper we discuss the distribution of polyphenols and minerals (Na, K, Mg, Ca, Al, P, S, Cr, Mn, Fe, Cu, Zn, Mo, As and Pb) in different seed parts (the rhizome, pericarp, carpel, seed coat and kernel) of the above species and their possible use in the nutritional supplements industry. The concentrations of total polyphenols, flavonoids and minerals ranged from 407 to 3144 mg rutin hydrate/100 g, 24 to 3070 mg quercetin/100 g and 1433 to 7928 mg/100 g, respectively. K, Ca, P and S were abundant in these herbal fruits. In two species of herbal fruits, *Terminalia arjuna* and *Terminalia chebula*, only part of the seed structure was suitable for use in nutritional supplements.

## 1. Introduction

Seeds contain vital nutrients and ultra-trace elements, which reduce the risk of cardiovascular disease and diabetes [1] and promote different healthy functions in human beings [2,3]. Many plants also contain polyphenols and flavonoids with strong antioxidant and disease-preventing properties, and could be valuable sources of these compounds in the preparation of nutritional supplements [4,5,6,7].

*Ziziphus mauritiana* (as *Ziziphus jujuba* (L.) Gaertn., and *Ziziphus jujuba* (L.) Lam.) is widely cultivated, especially in southeastern Asia, as a commercial crop [8]. The fruit is eaten raw or preserved and its seeds contain a number of medically active compounds, including saponins, triterpenes, flavonoids and alkaloids. It is hypnotic, narcotic, sedative, stomachic and tonic, and is used internally in the treatment of palpitations, insomnia, nervous exhaustion, night sweats and excessive perspiration [9]. *Buchanania lanzan* is a medium-sized deciduous tree with edible fruits and seed kernels. Its seed kernel and extracted kernel oil are used in the preparation of several Indian dishes and are a potential source of phytochemicals, tocopherols and essential fatty acids including oleic, linoleic and linolenic acid [10].

*Nilumbo nucifera* (Lotus) is an aquatic plant grown in tropical climates. Its rhizome and seeds are edible and have various therapeutic benefits including allelopathic, antiobesity, anti-HIV, antioxidant, diuretic, astringent, anti-inflammatory, hepatoprotective, antipyretic, antibacterial and immunomodulatory effects [11].

The Terminalia family (*Terminalia arjuna*, *Terminalia bellerica* and *Terminalia chebula*) includes several medicinal plants which have astringent and purgative properties and are used to treat cough, diarrhea, dropsy and leprosy, among other diseases [12].

Cucurbitaceae, of considerable economic importance, is a major source of food and medicine [13,14].

Studies have detected valuable polyphenols and minerals in some of these fruits [15,16,17,18,19,20,21,22], but no information is available as to their quantity and distribution within the pericarp, seed and seed coat.

Our study, therefore, was conducted to determine the quantity and distribution of total polyphenols, total flavonoids and minerals in the following widely-consumed seeds: *Buchanania lanzan*, *Ziziphus mauritiana*, *Nilumbo nucifera*, *Terminalia catappa*, *Terminalia arjuna*, *Terminalia bellirica*, *Terminalia chebula*, *Lagenaria siceraria*, *Luffa aegyptiaca*, *Praecitrullus fistulosus*, *Benincasa hispida*, *Citrullus lanatus* var. lanatus and *Cucurbita maxima*. Specifically, we determine the relative proportions of polyphenols and minerals in the pericarp, seed, seed coat and carpel of these representative species: *Buchanania lanzan*, *Ziziphus mauritiana*, *Nilumbo nucifera*, *Terminalia catappa*, *Terminalia arjuna*, *Terminalia bellirica*, *Terminalia chebula* and *Lagenaria siceraria*. Finally, we discuss the possibility of using these seeds as sources of polyphenols and minerals for nutritional supplements.

## 2. Results and Discussion

The mean values of each set of three analyses were calculated. The RSD for each group of data and species ranged from 0.68% to 24.0% and were considered homogeneous.

### 2.1. Physical Characteristics

Table 1 summarizes the physical characteristics of the seeds analyzed. The seed mass of the species *Buchanania lanzan*, Cucurbitaceae, *Nilumbo nucifera*, Terminalia (Combretaceae) and *Ziziphus mauritiana* ranged from 38 to 5770 mg per seed (*p* < 0.05). That of Terminalia was by far the highest. These seed masses were ordered as follows: Terminalia (4738 mg) > *Nilumbo nucifera* (1197 mg) > *Ziziphus mauritiana* (978 mg) > *Buchanania lanzan* (310 mg) > Cucurbitaceae seeds (126 mg).

**Figure 1:**
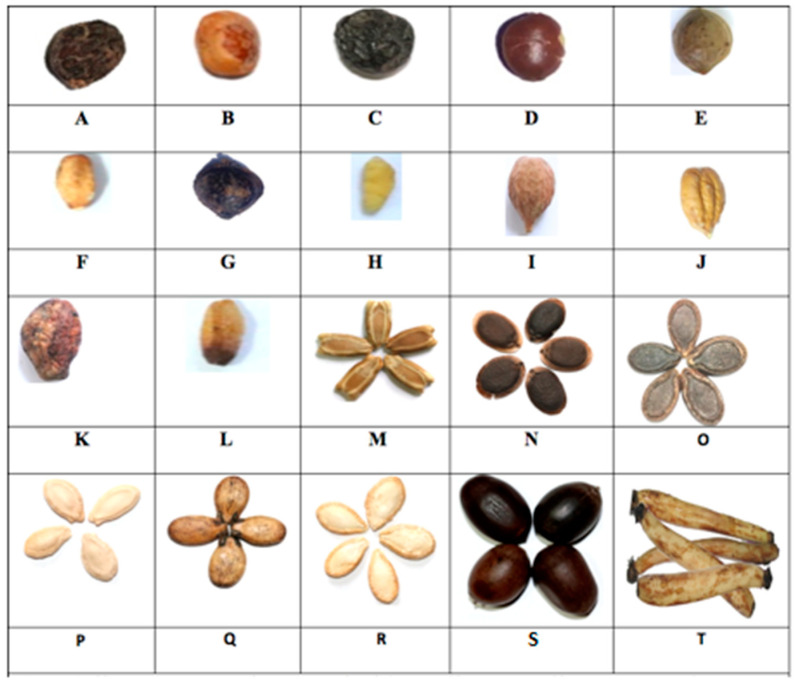
Seed images: (**A**) = *B. Lanzan* seed, (**B**) = *B. Lanzan* kernel, (**C**) = *Z. mauritiana*, (**D**) = *Z. mauritiana*, (**E**) = *T. catappa* seed, (**F**) = *T. catappa* kernel, (**G**) = *T Arjuna* seed, (**H**) = *T Arjuna* kernel, (**I**) = *T. Bellirica* seed, (**J**) = *T. Bellirica* kernel, (**K**) = *T. Chebula* seed, (**L**) = *T. Chebula* kernel, (**M**) = *L. siceraria* seed, (**N**) = *L. aegyptiaca* seed, (**O**) = *P. fistulosus* seed, (**P**) = *B. hispida* seed, (**Q**) = *C. lanatus* var. lanatus seed, (**R**) = *C. maxima* seed, (**S**) = *N. nucifera* seed, (**T**) = *N. nucifera* rhyzome.

The seeds were of diverse colors and shapes (Figure 1). The Buchanania lanzan, Terminalia and *Ziziphus mauritiana* seeds were composed of the pericarp, the hard seed coat and the kernel. The seeds of the other species were covered with a soft seed coat. The Cucurbitaceae seeds were covered with a large edible carpel.

The major fractions of the seeds were composed of the pericarp and the hard seed coat. The kernel masses accounted for 5.4% to 43% of the total (*p* < 0.05), in the following order: Cucurbitaceae seeds (43%) > *Buchanania lanzan* (24%) > Terminalia seeds (6.4%) > *Ziziphus mauritiana* (6.1%) > *Nilumbo nucifera* (5.4%).

The moisture content of the cultivars ranged from 1.2% for the *Ziziphus mauritiana* kernel to 13.5% for the Lagenaria siceraria carpel, with a mean value of 6.0% (*p* < 0.05).

### 2.2. Total Polyphenol (TPh) and Mineral Contents

Polyphenols are secondary metabolites that are synthesized by plants to protect against UV light, pathogens and herbivores [23]. Among several hundred polyphenols that have been detected in edible plants are flavonoids (Fla), one of the strongest and most abundant sources of antioxidants.

The concentration of total polyphenols (TPh) in the twelve seed kernels (KE) and six pericarps with seed coat (PC + SC) included in our analysis ranged from 407 to 2554 mg rutin hydrate/100 g (*p* < 0.05) and 1094 to 3144 mg rutin hydrate/100 g (*p* < 0.05), respectively (Table 2). As expected, the highest average content was found in the PC + SC samples (2242 mg rutin hydrate/100 g). Although the seed coat (SC) of *Lagenaria siceraria* also presented interesting values (1460 mg rutin hydrate/100 g), the fact that only two samples were available for analysis limited the value of the conclusions drawn. However, this was not the case of the KE samples, which also presented an interesting average total polyphenol content (1257 mg rutin hydrate/100 g). Nevertheless, no significant statistical differences (*p* > 0.05) were observed in the TPh contents of the different fruits according to the part of the plant analyzed (PC + SC, KE, SC, CA, SE, Rh).

**Figure 2:**
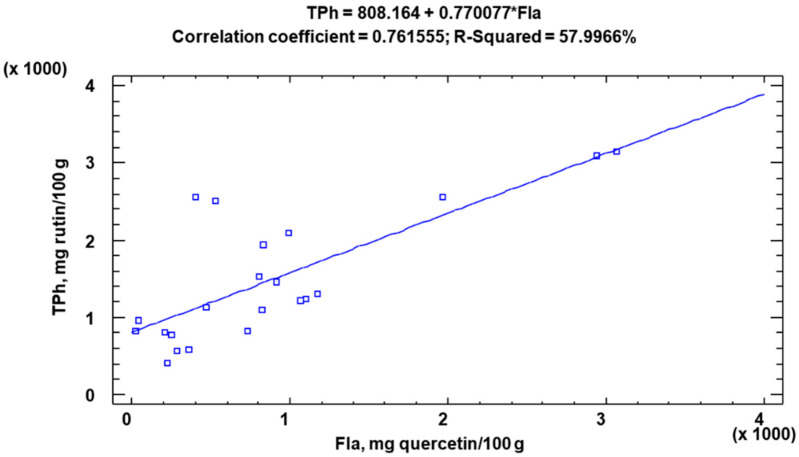
Correlation study between total Polyphenols (TPh) and Flavonoids (Fla).

The concentration of Fla in the KE and PC + SC samples ranged from 228 to 1970 mg quercetin/100 g (*p* < 0.05) and 525 to 3070 mg quercetin/100 g (*p* < 0.05), respectively (Table 2). The Fla presented a distribution similar to that found for total polyphenols, i.e., a higher content in the PC + SC samples (average 1526 mg quercetin/100 g), a high content in *Lagenaria siceraria* (SC) with 915 mg quercetin/100 g and high values of Fla in the KE samples (738 mg quercetin/100 g). This similarity in the distributions of the TPh and Fla contents seems to indicate a relationship between them, which would mean that Fla are the predominant polyphenolic species. This hypothesis was confirmed by a Pearson’s correlation study (Figure 2). The R-squared statistic indicates that the adjusted model explains 57.9966% of the variability in TPh. As expected, and so appears in many other studies, the correlation coefficient is equal to 0.761555, indicating a strong relationship between the variables.

The maximum TPh and Fla values were observed for the *Terminalia chebula* and *Terminalia arjuna* samples, with 2819 and 2543 mg rutin hydrate/100 g, respectively for TPh, and 2456 and 1950 mg quercetin/100 g, respectively for Fla. However, the ANOVA test did not reveal significant statistical differences between the different fruit species (*p* > 0.05). The highest TPh and Fla contents were recorded for the Combretaceae and Anacardiaceae and for the Combretaceae and Rhamnaceae families, respectively (*p* > 0.05). Comparable TPh contents in the Rhamnaceae, Cucurbitaceae and Combretaceae species have been detected in previous research [15,16,17,18,19].

Table 3 shows the concentrations of the minerals analyzed in the samples, which ranged from 1432.59 to 7927.99 mg/100 g (*p* < 0.05), with a mean value of 3730.24 mg/100 g. The highest values were observed for *Buchanania lanzan* (PC + SC), *Terminalia arjuna* (KE) and *Terminalia catappa* (KE). Comparable mineral contents were observed in the Terminalia species, analyzed by X-ray fluorescence [19]. However, markedly different mineral contents were observed in the African Cucurbitaceae species, possibly due to climatic variations [20,21,22].

The polyphenol and mineral contents of the samples were determined separately, in the pericarp (PC + SC) and in the seed kernel (KE), for *Buchanania lanzan*, *Ziziphus mauritiana*, *Nilumbo nucifera*, *Terminalia catappa*, *Terminalia arjuna*, *Terminalia bellirica*, *Terminalia chebula* and *Lagenaria siceraria*. Remarkably high levels of TPh were detected in the pericarp of four Terminalia species, ranging from 2055 to 3144 mg rutin hydrate/100 g. In contrast, the mineral contents were high in the seed kernels (4145 to 7928 mg/100 g) in all samples except *Terminalia bellirica*, in which the contents were similar in both phases. Higher TPh contents were also observed in the seed kernel phase of the following species: *Buchanania lanzan*, *Ziziphus mauritiana*, *Nilumbo nucifera* and *Lagenaria siceraria*. The mineral contents were much higher (4038 to 5271 mg/100 g) in the pericarp of *Buchanania lanzan* and *Nilumbo nucifera*. Finally, in *Terminalia bellirica* and *Lagenaria siceraria* the mineral content was similar in both phases.

### 2.3. Detailed Description of the Minerals

Potassium, an element promoting muscle strength, metabolism, water balance, electrolytic functions and the nervous system [24] was found in all of the species considered and in all parts of their fruits, ranging from 701.81 to 3427.72 mg/100 g, with a mean value of 1690.32 mg/100 g. The highest value was found for *Terminalia arjuna*, but there were no significant statistical differences between species or between different parts of the fruit (*p* > 0.05). On the other hand, the K content was significantly correlated with Ca in the kernel samples (KE) (r = 0.90).

Magnesium and calcium each play a major role in strengthening and maintaining the bones, muscles and nerves, and also contribute to protein synthesis and cellular metabolism [25]. In our samples, Mg was present in all species and in all parts of the fruits [26], ranging from 52.89 to 1455.81 mg/100 g, with a mean value of 496.50 mg/100 g. The highest value was found in *Praecitrullus fistulosus* (853 mg/100 g). In general, levels of Mg were higher in the kernel (KE) (mean value 769 mg/100 g) than elsewhere (mean value 169 mg/100 g). The large difference between KE and all other areas of the plant (PC + SC, CA, SC, SE, Rh) explains the significant statistical differences found according to the part of the plant (*p* < 0.05). The content of Mg was strongly correlated with that of P (r = 0.71–0.80).

The Ca contents in the pericarp (PC + SC), carpel (CA), rhizome (Rh) and kernel (KE) ranged from 32.34 to 3662.57 mg/100 g, with a mean value of 515.05 mg/100 g. Although the highest values were found in fruits with pericarp (PC + SC), 952.12 mg/100 g, there were no statistically significant differences between these concentrations and those in other types of fruits (*p* > 0.05). The highest values for Mg and Ca were recorded for the *Buchanania lanzan* pericarp and the *Terminalia arjuna* kernel, respectively. The Ca/K ratio ranged from 0.03 to 5.22, with a mean value of 0.61. This ratio was especially high in the *Buchanania lanzan* pericarp.

The Mg/Ca mass ratio ranged from 0.16 to 0.91 and 0.45 to 26.01, with mean values of 0.47 and 10.04 in the pericarp/carpel/seed cat/rhizome and seed kernel samples, respectively. These values are within the margins established as optimal for this relationship [27].

The phosphorus content in the pericarp/carpel/rhizome and kernel (PC + SC, CA, Rh, KE) ranged from 25.95 to 1617.28 mg/100 g, with a mean value of 680.67 mg/100 g. In this case, the differences between the fruits were statistically significant (*p* < 0.05). These data are similar to those found for other plant species [28]

The concentration of sulphur in the samples ranged from 32.28 to 367.84 mg/100 g in the pericarp/carpel/rhizome and in the kernel, respectively (*p* < 0.05), with an average value of 209.64 mg/100 g. The highest P and S contents in the pericarp were recorded in the *Terminalia chebula* carpel. In the kernel, the highest P and S values were obtained for *Terminalia arjuna* and *Citrullus lanatus* var. lanatus. P and S were strongly correlated (r = 0.85–0.99) in all the samples. The S/P mass ratio ranged from 0.21 to 1.35, with a mean value of 0.54. The highest S/P mass ratios were observed in the Terminalia pericarp (PC + SC), ranging from 0.98 to 1.35.

In the analyzed samples, the content of sodium, which plays a key role in osmoregulation and fluid maintenance in the human body, ranged from 2.31 to 1549.87 mg/100 g, with a mean value of 83.76 mg/100 g for the pericarp/carpel/rhizome and kernel (PC + SC, CA, Rh, KE), with no significant statistical differences between these areas (*p* > 0.05). A very high value (1549.87 mg/100 g) was detected for the *Terminalia catappa* kernel. There was a strong correlation (r = 0.78–1.0) between Na and Al, Pb and Zn in the fruits with kernel.

Iron, which is essential to metabolize proteins and in the production of hemoglobin and red blood cells [25], was present in low concentrations in the pericarp/carpel/rhizome and seed kernels (PC + SC, CA, Rh, KE), ranging from 4.77 to 64.64 mg/100 g (*p* > 0.05) with a mean value of 13.16 mg/100 g. However, there was a high Fe content in the *Nilumbo nucifera* rhizome (64.64 mg/100 g), followed by the *Terminalia arjuna* (35.84 mg/100 g) and the *Buchanania lanzan* (22.57 mg/100 g) pericarp. The Fe content correlated strongly (r = 0.81–0.98) with that of Al and Mn.

The content of manganese, another essential element, which promotes the growth of bone structures [24], ranged from 0.49 to 8.69 mg/100 g (*p* > 0.05), with a mean value of 3.40 mg/100 g. The highest values were recorded in the *Nilumbo nucifera* rhizome and seed. The Mn/Fe mass ratio ranged from 0.05 to 0.75, with a mean value of 0.33.

Copper, vital for a range of body functions including the production of red blood cells and energy and the maintenance of nerve cells and the immune system [25] was detected in 17 of the samples, at concentrations ranging from 0.27 to 181.64 mg/100 g (*p* < 0.05), with a mean value of 13.48 mg/100 g. The highest contents were detected in the *Terminalia arjuna* pericarp (181.64 mg/100 g) and in the *Terminalia catappa* pericarp (18.96 mg/100 g). The Cu concentration observed in the *Terminalia arjuna* pericarp was similar to that reported elsewhere [19].

Zinc, essential to maintain the immune and digestive systems [24] was present in most of the seed kernel samples (KE). The lowest levels were recorded in *Luffa aegyptiaca* (0.57 mg/100 g) and the highest in *Terminalia catappa* (10.76 mg/100 g). These high levels of Zn are appropriate due to the usual deficiency of this mineral in India [29]. Statistically significant differences (*p* < 0.05) were observed among the Zn concentrations in the kernel samples. There were strong correlations (0.75–0.89) between Zn and Al, Cr, Na and Pb.

Chromium is another essential trace mineral, which heightens insulin sensitivity and promotes the metabolism of proteins, carbohydrates and lipids [24]. Our analysis detected Cr in fourteen samples, corresponding to *Buchanania lanzan*, *Cucurbita maxima*, *Ziziphus mauritiana*, *Nilumbo nucifera*, *Lagenaria siceraria*, *Luffa aegyptiaca* and Terminalia seeds, at concentrations ranging from 0.02 to 1.41 mg/100 g, with no significant statistical differences (*p* > 0.05). The content was appreciable in the *Buchanania lanzan* pericarp (PC + SC), at 1.41 mg/100 g.

In fifteen samples, nickel was detected at trace levels, ranging from 0.01 to 0.95 mg/100 g (*p* > 0.05). The highest value was recorded for the *Citrullus lanatus* (KE), followed by *Terminalia arjuna* (KE) and *Terminalia catappa* (KE) (0.86 and 0.82 mg/100 g, respectively).

Molybdenum participates in enzymatic systems and is related to the metabolism of uric acid, alcohol, drugs, sulphites and toxins, among others [24]. A small amount (0.05 mg/100 g) was detected in one sample, *Terminalia bellirica* (PC + SC).

Aluminum is a non-essential element. Nevertheless, it was detected in all samples, at concentrations ranging from 0.45 to 388.17 mg/100 g (*p* > 0.05). Particularly high contents were observed in the *Terminalia catappa* kernel sample (388.17 mg/100 g), the *Nilumbo nucifera* rhizome (57.81 mg/100 g) and the *Terminalia arjuna* pericarp (30.42 mg/100 g). Although these concentrations seem high, Al is unlikely to be a human carcinogen at normal dietary doses [30]. The content of Al was strongly correlated (r = 0.70–1.0) with Na, Pb and Zn (KE), and with Fe and Mn (Rh/PC + SC).

Lead, a toxic element, was found in nine samples belonging to the Combretaceae, Cucurbitaceae, Nelumbonaceae and Rhamnaceae families, with a content ranging from 0.22 to 2.94 mg/100 g (*p* > 0.05). In Cucurbitaceae and Combretaceae, this range was 0.29 to 2.94 mg/100 g. In five samples, *Terminalia catappa* (average concentration 2.72 mg/100 g), *Terminalia arjuna* (1.76 mg/100 g), *Terminalia bellirica* (1.30 mg/100 g) and *Ziziphus mauritiana* (0.53 mg/100 g), the content exceeded the permissible limit of 0.5 mg/100 g [31].

Another toxic element, arsenic, was detected in three Cucurbitaceae species: *Lagenaria siceraria* (CA), *Benincasa hispida* (KE), *Luffa aegyptiaca* (KE), with a content ranging from 1.27 to 4.75 mg/100 g (*p* > 0.05).

These two minerals are highly undesirable in human nutrition and so the following species should be excluded from consideration: *Ziziphus mauritiana* (KE), *Nilumbo nucifera* (Rh), *Terminalia catappa* (PC + SC, KE), *Terminalia arjuna* (KE), *Terminalia bellirica* (PC + SC), *Lagenaria siceraria* (CA, KE), *Luffa aegyptiaca* (KE), *Benincasa hispida* (KE) and *Citrullus lanatus* var. lanatus (KE). Table 4 lists the species in which lead and/or arsenic were detected.

### 2.4. Viability of Herbal Fruits as a Source of Polyphenols and Minerals for Nutritional Supplements

**Figure 3:**
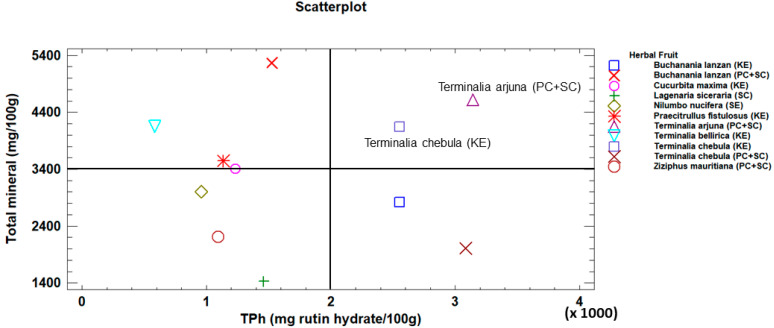
Scatterplot for Total Polyphenols (TPh) and total mineral content.

Having discarded the samples containing lead and/or arsenic, we then analyzed the remainder to determine which would be most suitable as a source of polyphenols and minerals. The TPh value was taken as a reference for this purpose, since the Fla content was closely related to that of total polyphenols. The content of total minerals was also considered, as most of these minerals were present in the viable samples (Figure 3 and Figure S1a,b). This analysis showed that two samples in particular, *Terminalia arjuna* (PC + SC) and *Terminalia chebula* (KE) were highly suitable as sources of TPh and minerals in the preparation of nutritional supplements.

## 3. Materials and Methods

### 3.1. Sampling of Herbal Fruits

The above-mentioned herbal fruits were collected from Raipur (India) during May-June 2017 (summer), in accordance with ISO recommendations [32]. The fruits were sun dried for one week indoors, under glass and on a glass plate. The *Lagenaria siceraria* pericarp and seeds were separated manually before drying. The seeds were also oven-dried overnight at 50 °C.

### 3.2. Sample Preparation

In every case, the pericarp, seed, seed coat and kernel were separated from the fruit. They were then crushed to a powder, sieved to obtain particles of ≤0.1 mm and stored in glass containers. The mass of each cultivar was measured using a Mettler electronic balance. Composite samples were prepared by mixing three samples in identical mass ratios. The moisture content of the sample was determined by heating at 105 °C for 6 h.

### 3.3. Analysis of Polyphenols

For each analysis, 100 mg of sample was extracted with 5 mL of an acetone:water (70:30, *v*/*v*) solution maintained in an ultra-sonic bath for 20 min at 20 °C. Then, 5 mL of fresh acetone: water (70:30, *v*/*v*) solution was added to the mixture and the extraction was repeated for 20 min at 20 °C. After centrifugation, the supernatant was collected [33]. The total phenolic content (TPh) of each extract was determined as rutin hydrate, using the Folin-Ciocalteu reagent and according to the method of Singleton, Orthofer, and Lamuela-Raventós [34,35]. The flavonoid (Fla) content was determined by the aluminum chloride method as quercetin [36]. Each analysis was conducted in triplicate.

### 3.4. Analysis of Minerals

A precisely-weighed amount (0.10 g) of sample was mixed with 5.0 mL of the acid solution in a Teflon tube for microwave digestion. The solution was diluted to 100 mL with deionized water for the ICP-OES and ICP-MS analysis of the mineral, using appropriate standard reference materials [37].

### 3.5. Statistics

The Statgraphics^®^ Centurion XVI (v16. StatPoint Technologies, Inc., Warrenton, VA, USA) statistical package was used to interpret the data obtained. The coefficients of variation (CV) or relative standard deviations (RSD) for each group of data and species were calculated, to determine their distributive characteristics and to enable appropriate statistical tests (parametric or non-parametric)—ANOVA, Bartlett, Kruskal-Wallis, Student’s *t*-test or Pearson’s correlation—to be applied. Statistical significance was assumed at *p* ≤ 0.05.

## 4. Conclusions

The species *Terminalia arjuna*, *Buchanania lanzan* and *Nilumbo nucifera* pericarp/rhizome contain high levels of minerals and polyphenols. The ripe fruit of *Buchanania lanzan* is a good source of polyphenols and Mg, Ca and Cr. *Nilumbo nucifera* rhizome is a good source of the polyphenols and of P, K. Fe and Mn. *Terminalia arjuna* contained useful levels of polyphenols and of P, K, S, Mg and Cu. *Terminalia catappa* was a significant source of polyphenols and of Al, Na and Zn. Within the Curcutbitaceae family, *Citrullus lanatus* var. lanatus, the seed kernel was a potential source of polyphenols and of Ni, S and Mg. However, apart from the levels of polyphenols and minerals recorded, only *Terminalia arjuna* (PC + SC) and *Terminalia chebula* (KE) presented the ideal conditions for the elaboration of nutritional supplements.

## Figures and Tables

**Table 1:** Physico-chemical characteristics of herbal fruits.

Species	Family	Part	Seed Mass (mg)	Kernel Mass (%)	Moisture (%)
*Buchanania lanzan*	Anacardiaceae	PC + SC	310.0	24.0	5.2
*Buchanania lanzan*	Anacardiaceae	KE	-	-	1.9
*Ziziphus mauritiana*	Rhamnaceae	PC + SC	978.0	6.1	6.1
*Ziziphus mauritiana*	Rhamnaceae	KE	-	-	1.2
*Nilumbonucifera* -I	Nelumbonaceae	Rh	-	10.5	10.5
*Nilumbo nucifera* -II	Nelumbonaceae	Rh	-	10.4	10.4
*Nilumbo nucifera* -I	Nelumbonaceae	SE	1213.0	5.4	7.2
*Nilumbo nucifera* -II	Nelumbonaceae	SE	1180.0	5.3	6.9
*Terminaliacatappa* -I	Combretaceae	PC + SC	4763.0	8.3	4.8
*Terminalia catappa* -II	Combretaceae	PC + SC	5770.0	8.4	4.3
*Terminalia catappa* -I	Combretaceae	KE	-	-	2.8
*Terminalia catappa* -II	Combretaceae	KE	-	-	2.2
*Terminalia arjuna*	Combretaceae	PC + SC	3885.0	3.1	4.2
*Terminalia arjuna*	Combretaceae	KE	-	-	3.8
*Terminalia bellirica*	Combretaceae	PC + SC	4373.0	11.0	5.2
*Terminalia bellirica*	Combretaceae	KE	-	-	3.5
*Terminalia chebula*	Combretaceae	PC + SC	5426.0	3.1	4.9
*Terminalia chebula*	Combretaceae	KE	-	-	3.0
*Lagenaria siceraria* -I	Cucurbitaceae	CA	-	-	12.6
*Lagenaria siceraria* -II	Cucurbitaceae	CA	-	-	13.5
*Lagenaria siceraria* -I	Cucurbitaceae	SC	240.0	47.0	4.5
*Lagenaria siceraria* -II	Cucurbitaceae	SC	216.0	58.0	5.0
*Lagenaria siceraria* -I	Cucurbitaceae	KE	-	-	4.6
*Lagenaria siceraria* -II	Cucurbitaceae	KE	-	-	4.4
*Luffa aegyptiaca*	Cucurbitaceae	KE	105.0	43.0	5.5
*Praecitrullus fistulosus*	Cucurbitaceae	KE	90.0	46.0	9.6
*Benincasa hispida*	Cucurbitaceae	KE	64.0	47.0	8.3
*Citrullus lanatus* var. lanatus	Cucurbitaceae	KE	38.0	49.0	6.5
*Cucurbita maxima*	Cucurbitaceae	KE	132.0	18.0	7.8

**Table 2:** Polyphenol content of herbal fruits.

Species	Part	TPh mg Rutin Hydrate/100 g	Fla mg Quercetin/100 g
*Buchanania lanzan*	PC + SC	1525.0 ± 10.44	805.0 ± 8.72
*Buchanania lanzan*	KE	2553.0 ± 13.53	406.0 ± 5.57
*Ziziphus mauritiana*	PC + SC	1094.0 ± 15.10	820.0 ± 6.24
*Ziziphus mauritiana*	KE	1210.0 ± 6.24	1065.0 ± 6.08
*Nilumbo nucifera* -I	Rh	778.0 ± 12.53	20.0 ± 2.65
*Nilumbo nucifera* -II	Rh	857.0 ± 11.27	28.0 ± 3.61
*Nilumbo nucifera* -I	SE	883.0 ± 7.55	41.0 ± 2.65
*Nilumbo nucifera* -II	SE	1030.0 ± 7.81	44.0 ± 3.61
*Terminalia catappa* -I	PC + SC	2219.0 ± 7.94	1053.0 ± 7.55
*Terminalia catappa* -II	PC + SC	1978.0 ± 11.36	932.0 ± 5.57
*Terminalia catappa* -I	KE	576.0 ± 6.56	300.0 ± 6.93
*Terminalia catappa* -II	KE	544.0 ± 6.24	265.0 ± 4.58
*Terminalia arjuna*	PC + SC	3144.0 ± 8.19	3070.0 ± 13.08
*Terminalia arjuna*	KE	1943.0 ± 9.85	830.0 ± 6.56
*Terminalia bellirica*	PC + SC	2505.0 ± 8.66	525.0 ± 5.29
*Terminalia bellirica*	KE	584.0 ± 4.58	360.0 ± 4.58
*Terminalia chebula*	PC + SC	3085.0 ± 13.89	2942.0 ± 11.27
*Terminalia chebula*	KE	2554.0 ± 9.54	1970.0 ± 9.64
*Lagenaria siceraria* -I	CA	831.0 ± 7.94	214.0 ± 3.46
*Lagenaria siceraria* -II	CA	778.0 ± 7.55	208.0 ± 3.61
*Lagenaria siceraria* -I	SC	1480.0 ± 9.64	939.0 ± 9.85
*Lagenaria siceraria* -II	SC	1440.0 ± 8.72	890.0 ± 8.54
*Lagenaria siceraria* -I	KE	1340.0 ± 7.21	1205.0 ± 7.94
*Lagenaria siceraria* -II	KE	1256.0 ± 10.58	1140.0 ± 6.56
*Luffa aegyptiaca*	KE	777.0 ± 6.56	252.0 ± 3.46
*Praecitrullus fistulosus*	KE	1136.0 ± 9.64	466.0 ± 4.58
*Benincasa hispida*	KE	407.0 ± 5.57	228.0 ± 4.36
*Citrullus lanatus* var. lanatus	KE	828.0 ± 8.19	728.0 ± 6.24
*Cucurbita maxima*	KE	1230.0 ± 8.89	1100.0 ± 9.54

**Table 3:** Mineral content of herbal fruit, mg/100 g.

Species	Part	Al	Ca	Cr	Cu	Fe ^(^**^)^	K	Mg ^(^**^)^	Mn
BL	PC + SC	5.17 ± 0.08	3662.57 ± 3.01	1.41 ± 0.19	8.44 ± 0.37	22.57 ± 0.19	701.81 ± 3.87 ^ϕ^	602.93 ± 6.28	6.72 ± 0.08
BL	KE	6.03 ± 0.24	122.25 ± 0.27	0	0.75 ± 0.06	8.59 ± 0.38	932.57 ± 2.90 ^ϕ^	517.21 ± 3.30	2.25 ± 0.10
ZM	PC + SC	1.91 ± 0.17	220.58 ± 0.14	0.25 ± 0.07	0	6.52 ± 0.19	1656.54 ± 11.04	142.46 ± 2.34	1.16 ± 0.12 ^√^
ZM	KE	0.73 ± 0.15	145.96 ± 0.14	0.31 ± 0.05	0.27 ± 0.04	9.13 ± 0.12	991.93 ± 2.69	421.96 ± 3.59	2.94 ± 0.11 ^√^
NN	Rh	57.81 ± 2.30	168.74 ± 0.14	0.11 ± 0.03	0	64.64 ± 0.13	2983.08 ± 7.54	101.92 ± 3.40	8.69 ± 0.17 ^ϕ®£¥∞©√^
NN	SE	8.67 ± 0.34	184.03 ± 0.09	0	0.66 ± 0.08	11.12 ± 0.93	1626.71 ± 6.12	172.75 ± 6.91	6.54 ± 0.26 ^ϕ®£¥∞©√^
TC	PC + SC	8.36 ± 0.33 ^ϕ^	183.87 ± 0.06	0	18.96 ± 0.80	13.39 ± 0.35	3231.41 ± 12.53	61.31 ± 3.05	0.85 ± 0.16 ^∞^
TC	KE	388.17 ± 5.46 ^ϕ^	473.05 ± 0.20	0.39 ± 0.03	1.98 ± 0.13	12.86 ± 0.75	1484.08 ± 15.52	664.31 ± 3.23	2.27 ± 0.11 ^∞^
TA	PC + SC	30.42 ± 1.41	582.98 ± 0.20	0.13 ± 0.04	181.64 ± 0.95 ^ϕ^	35.84 ± 0.97	3427.72 ± 25.25 ^ϕ^	185.58 ± 4.39	4.12 ± 0.10
TA	KE	1.08 ± 0.07	1874.19 ± 1.23	0.10 ± 0.02	3.22 ± 0.10 ^ϕ^	8.01 ± 0.39	2583.91 ± 35.78 ^ϕ^	1455.81 ± 9.44	5.47 ± 0.13
TB	PC + SC	4.35 ± 0.17	982.94 ± 1.16	0	0	11.45 ± 0.29	2969.25 ± 31.13	174.46 ± 2.15	2.79 ± 0.12 ^¥^
TB	KE	1.02 ± 0.06	1195.66 ± 1.25	0.12 ± 0.02	1.79 ± 0.20	4.86 ± 0.27	1391.55 ± 35.95	532.91 ± 4.51	1.41 ± 0.08 ^¥^
TCH	PC + SC	1.75 ± 0.13	79.77 ± 0.32	0.10 ± 0.03	<	9.00 ± 0.29	1797.79 ± 11.84	57.92 ± 5.13	0.49 ± 0.13 ^©^
TCH	KE	1.75 ± 0.15	533.23 ± 0.20	0.14 ± 0.04	2.39 ± 0.08	5.74 ± 0.27	1379.31 ± 10.01	970.77 ± 9.58	3.83 ± 0.15 ^©^
LS	CA	4.98 ± 0.15 ^ϕ^	424.48 ± 0.16	0.11 ± 0.04	0 ^ϕ^	9.55 ± 0.41	2549.35 ± 10.71	140.05 ± 4.31	2.06 ± 0.12 ^ϕ^
LS	SC	3.65 ± 0.11 ^ϕ^	58.03 ± 0.12	0.02 ± 0.02	1.04 ± 0.08 ^ϕ^	5.96 ± 0.32	1175.24 ± 6.01	52.89 ± 2.01	1.72 ± 0.10 ^ϕ^
LS	KE	1.03 ± 0.03 ^ϕ^	50.38 ± 0.15	0	4.35 ± 0.09 ^ϕ^	9.35 ± 0.13	942.15 ± 5.33	798.03 ± 8.22	3.24 ± 0.12 ^ϕ^
LA	KE	0.45 ± 0.02	33.81 ± 0.11	0.15 ± 0.06	0.51 ± 0.06	4.77 ± 0.57	1312.13 ± 9.91	709.21 ± 7.31	2.04 ± 0.11 ^®^
PF	KE	2.29 ± 0.07	172.19 ± 0.10	0	0.68 ± 0.05	11.47 ± 0.27	1048.62 ± 14.42	853.17 ± 6.82	2.23 ± 0.08 ^£^
BH	KE	4.83 ± 0.15	81.24 ± 0.24	0	0.76 ± 0.39	7.84 ± 0.71	954.82 ± 11.19	786.11 ± 9.98	5.85 ± 0.23
CL	KE	4.95 ± 0.15	68.74 ± 0.26	0	0.93 ± 0.07	7.77 ± 0.19	1054.87 ± 13.15	680.03 ± 6.26	3.21 ± 0.12
CM	KE	0.70 ± 0.11	32.34 ± 0.09	0.02 ± 0.01	0.76 ± 0.08	9.04 ± 0.64	992.10 ± 18.35	841.09 ± 7.87	4.89 ± 0.20
**Species**	**Part**	**Mo**	**Na**	**Ni**	P ^(^**^)^	Pb ^(^*^)^	S ^(^**^)^	**Zn**	As ^(^*^)^
BL	PC + SC	0	14.90 ± 0.32	0.23 ± 0.03	130.91 ± 0.35	0	113.00 ± 0.28	0	0
BL	KE	0	7.94 ± 0.10	0.27 ± 0.04	936.12 ± 0.81	0	285.89 ± 3.94	2.19 ± 0.07	0
ZM	PC + SC	0	4.23 ± 0.07 ^¥^	0 ^®^	111.64 ± 1.41	0	63.47 ± 0.46	0	0
ZM	KE	0	4.75 ± 0.05 ^¥^	0 ^®^	870.25 ± 2.22	0.53 ± 0.07	337.09 ± 2.80	4.82 ± 0.13	0
NN	Rh	0	51.63 ± 0.25	0.17 ± 0.04	404.67 ± 1.52	0.22 ± 0.03	196.78 ± 0.31	0	0
NN	SE	0	9.66 ± 0.10	0.50 ± 0.05	743.22 ± 2.27	0	237.51 ± 1.36	0	0
TC	PC + SC	0	60.01 ± 0.96 ^ϕ®£¥^	0.01 ± 0.01	78.03 ± 0.76	2.94 ± 0.08	72.40 ± 0.65	0	0
TC	KE	0	1549.87 ± 12.94 ^ϕ®£¥^	0.82 ± 0.03	1193.62 ± 4.24	2.49 ± 0.09	251.42 ± 0.57	10.76 ± 0.10	0
TA	PC + SC	0	22.39 ± 0.18	0.52 ± 0.09	64.15 ± 1.09	0	86.50 ± 0.55	0	0
TA	KE	0	7.78 ± 0.12	0.86 ± 0.05	1617.28 ± 3.25	1.76 ± 0.06	366.76 ± 0.56	1.76 ± 0.09	0
TB	PC + SC	0.05 ± 0.01	7.01 ± 0.13 ^®^	0.28 ± 0.02	59.74 ± 1.75	1.30 ± 0.04	74.07 ± 1.47	0	0
TB	KE	0	11.83 ± 0.16 ^®^	0.76 ± 0.04	771.51 ± 1.31	0	233.10 ± 1.23	0	0
TCH	PC + SC	<	2.31 ± 0.09 ^£^	<	25.95 ± 0.87	<	32.28 ± 0.77	<	<
TCH	KE	<	7.12 ± 0.11 ^£^	0.78 ± 0.06	897.02 ± 5.59	<	339.92 ± 0.88	3.47 ± 0.30	<
LS	CA	0	7.36 ± 0.08 ^ϕ^	0.29 ± 0.05 ^ϕ^	161.56 ± 0.39	0	80.81 ± 0.41	0	1.27 ± 0.04
LS	SC	0	6.64 ± 0.10 ^ϕ^	0 ^ϕ^	86.04 ± 0.60	0	41.36 ± 1.13	0	0
LS	KE	0	8.00 ± 0.11 ^ϕ^	0 ^ϕ^	1150.64 ± 1.52	0.29 ± 0.03	294.11 ± 0.48	1.71 ± 0.17	0
LA	KE	0	4.93 ± 0.07	0	1044.37 ± 1.19	0.33 ± 0.03	250.00 ± 0.64	0.57 ± 0.06	4.75 ± 0.05
PF	KE	0	18.59 ± 0.21	0.35 ± 0.10	1127.10 ± 2.01	0	307.79 ± 0.83	1.82 ± 0.06	0
BH	KE	0	8.82 ± 0.14	0.18 ± 0.05	1191.30 ± 5.48	0	292.87 ± 0.90	2.38 ± 0.08	1.86 ± 0.04
CL	KE	0	8.05 ± 0.11	0.95 ± 0.05 ^ϕ^	1091.43 ± 7.07	0.41 ± 0.04	367.84 ± 0.88	1.44 ± 0.10	0
CM	KE	0	18.81 ± 0.08	0	1218.13 ± 3.01	0	287.02 ± 0.91	2.19 ± 0.07	0

Data = Mean ± Sd. (*) indicates *p* ≤ 0.05 among all species. (**) indicates *p* ≤ 0.05 among all plant parts. The same superscript symbol indicates *p* ≤ 0.05 among specific species. BL = *Buchanania lanzan*, ZM = *Ziziphus mauritiana*, NN = *Nilumbo nucifera*, TC = *Terminalia catappa*, TA = *Terminalia arjuna*, TB = *Terminalia bellirica*, TCH= *Terminalia chebula*, LS = *Lagenaria siceraria*, LA = *Luffa aegyptiaca*, PF = *Praecitrullus fistulosus*, BH = *Benincasa hispida*, CL *= Citrullus lanatus var. lanatus*, CM = *Cucurbita máxima*, PC + SC = Pericarp + Seed Coat, SC = Seed Coat, KE = Kernel, CA = Carpel, SE = Seed, Rh = Rhyzome, < = below quantification limit.

**Table 4:** Viability of herbal fruits based on their lead and arsenic content.

Species	Part	Lead	Arsenic	Viability
*Buchanania lanzan*	PC + SC			yes
*Buchanania lanzan*	KE			yes
*Ziziphus mauritiana*	PC + SC			yes
*Ziziphus mauritiana*	KE	+		not
*Nilumbo nucifera* -I	Rh	+		not
*Nilumbo nucifera* -II	Rh	+		not
*Nilumbo nucifera* -I	SE			yes
*Nilumbo nucifera* -II	SE			yes
*Terminalia catappa* -I	PC + SC	+		not
*Terminalia catappa* -II	PC + SC	+		not
*Terminalia catappa* -I	KE	+		not
*Terminalia catappa* -II	KE	+		not
*Terminalia arjuna*	PC + SC			yes
*Terminalia arjuna*	KE	+		not
*Terminalia bellirica*	PC + SC	+		not
*Terminalia bellirica*	KE			yes
*Terminalia chebula*	PC + SC			yes
*Terminalia chebula*	KE			yes
*Lagenaria siceraria* -I	CA		+	not
*Lagenaria siceraria* -II	CA		+	not
*Lagenaria siceraria* -I	SC			yes
*Lagenaria siceraria* -II	SC			yes
*Lagenaria siceraria* -I	KE	+		not
*Lagenaria siceraria* -II	KE	+		not
*Luffa aegyptiaca*	KE	+	+	not
*Praecitrullus fistulosus*	KE			yes
*Benincasa hispida*	KE		+	not
*Citrullus lanatus* var. lanatus	KE	+		not
*Cucurbita maxima*	KE			yes

## Data Availability

All data are included along the text in the corresponding tables.

## Supplementary Materials

The following supporting information can be downloaded at: https://www.mdpi.com/article/10.3390/molecules28062682/s1, Figure S1. (a) Total Polyphenols (TPh). (b) Total mineral content comparison.

## Sample Availability

Original seeds are available upon request to Patel.
