# Supplementary material for: Comparative Analysis of Traditional Oriental Herbal Fruits as Potential Sources of Polyphenols and Minerals for Nutritional Supplements
Source: Molecules. 2023 Mar 16;28(6):2682. doi: 10.3390/molecules28062682 (PMC10058731; doi:10.3390/molecules28062682)
Supplement: Supplementary file 1 [file molecules-28-02682-s001.zip › molecules-2215148-supplementary.pdf]

Supplementary material

Comparative analysis of traditional oriental herbal fruits as potential sources of polyphenols and minerals for nutritional supplements

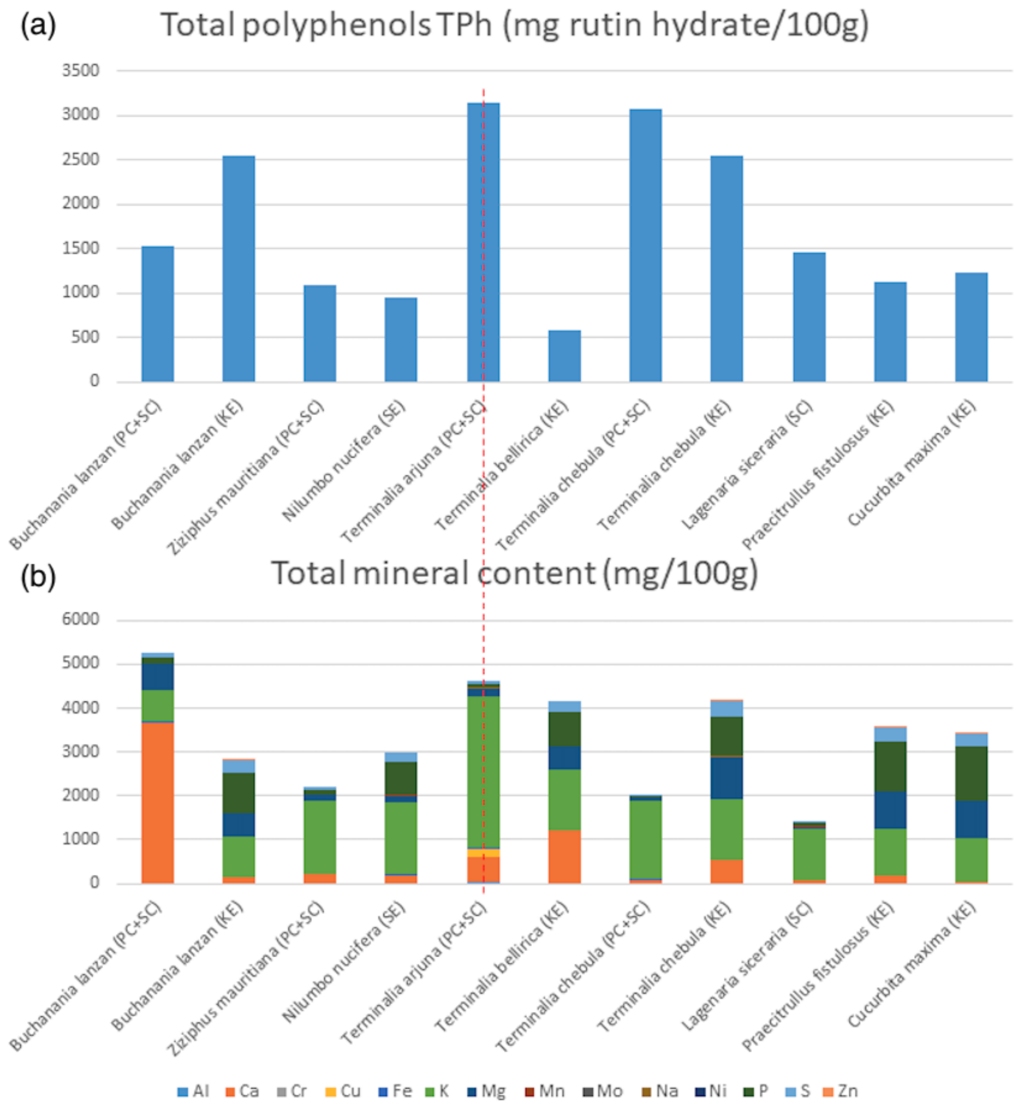

Figure S1. (a) Total Polyphenols (TPh). (b) Total mineral content comparison.
